# Domain-Specific Inhibitory Control Training to Improve Children’s Learning of Counterintuitive Concepts in Mathematics and Science

**DOI:** 10.1007/s41465-019-00161-4

**Published:** 2019-12-12

**Authors:** Hannah R. Wilkinson, Claire Smid, Su Morris, Emily K. Farran, Iroise Dumontheil, Sveta Mayer, Andrew Tolmie, Derek Bell, Kaśka Porayska-Pomsta, Wayne Holmes, Denis Mareschal, Michael S. C. Thomas, The UnLocke Team

**Affiliations:** 1 Centre for Brain and Cognitive Development, Birkbeck, University of London, London, UK; 2 Centre for Brain and Cognitive Development, Birkbeck, University of London, London, UK; 3 UCL Institute of Education, University College of London, London, UK; 4 School of Psychology, University of Surrey, Guildford, UK; 5 Learnus, London, UK; 6 Nesta, London, UK

**Keywords:** Cognitive training, Counterintuitive reasoning, Inhibitory control, Mathematics achievement, Misconceptions, Science achievement, Technology-enhanced learning

## Abstract

Evidence from cognitive neuroscience suggests that learning counterintuitive concepts in mathematics and science requires inhibitory control (IC). This prevents interference from misleading perceptual cues and naïve theories children have built from their experiences of the world. Here, we (1) investigate associations between IC, counterintuitive reasoning, and academic achievement and (2) evaluate a classroom-based computerised intervention, called Stop & Think, designed to embed IC training within the learning domain (i.e. mathematics and science content from the school curricula). Cross-sectional analyses of data from 627 children in Years 3 and 5 (7- to 10-year-olds) demonstrated that IC, measured on a Stroop-like task, was associated with counterintuitive reasoning and mathematics and science achievement. A subsample (*n* = 456) participated either in Stop & Think as a whole-class activity (teacher-led, STT) or using individual computers (pupil-led, STP), or had teaching as usual (TAU). For Year 3 children (but not Year 5), Stop & Think led to better counterintuitive reasoning (i.e. near transfer) in STT (*p* < .001, η_p_^2^ = .067) and STP (*p* < .01, η_p_^2^ = .041) compared to TAU. Achievement data was not available for Year 3 STP or Year 5 STT. For Year 3, STT led to better science achievement (i.e. far transfer) compared to TAU (*p* < .05, η_p_^2^ = .077). There was no transfer to the Stroop-like measure of IC. Overall, these findings support the idea that IC may contribute to counterintuitive reasoning and mathematics and science achievement. Further, we provide preliminary evidence of a domain-specific IC intervention with transferable benefits to academic achievement for Year 3 children.

**Electronic supplementary material:**

The online version of this article (10.1007/s41465-019-00161-4) contains supplementary material, which is available to authorized users.

## Introduction

A set of complex cognitive processes, or ‘executive functions’ (EFs), is required to direct behaviour, solve problems, and achieve goals (Blair, 2016; Diamond, 2013). These EFs include working memory (temporarily holding and manipulating information), cognitive flexibility (switching between tasks, strategies, or perspectives), and inhibitory control (IC; focusing attention and withholding impulsive responses) (Miyake et al., 2000). These skills are foundational for academic tasks and learning. EFs have been linked to academic achievement from preschool and throughout the school years, even after controlling for IQ (for reviews, see Allan et al., 2014; Donati et al., 2019; Jacob & Parkinson, 2015; Zelazo et al., 2016).

In particular, EFs have been associated with achievement in mathematics (e.g. Bull et al., 2008; Cragg & Gilmore, 2014; Friso-Van den Bos et al., 2013; Laski & Dulaney, 2015) and literacy (e.g. Blair & Razza, 2007; Christopher et al., 2012; Kieffer et al., 2013; Nouwens et al., 2016). The relationship between EFs and children’s science achievement has received less research attention (see Tolmie et al., 2016). However, there is evidence of a positive relationship. St Clair-Thompson and Gathercole (2006) found that IC in 10- to 11-year-olds was associated with English, mathematics and science achievement, and Nayfeld et al. (2013) found that executive functioning (an aggregate measure of working memory, cognitive flexibility, and IC) in 4-year-olds predicted preschool science achievement to a significantly greater degree than mathematics or literacy. While research examining EFs and science achievement is relatively limited, there is considerable evidence that suggests that EFs are involved in reasoning about both mathematics and science concepts. For example, Zaitchik et al. (2014) found an aggregate measure of executive functioning to be associated with 5- to 7-year-olds’ reasoning about biological processes of life, death and bodily functions. Recent research with both children and adolescents has found IC to be associated with performance on tasks comprising a range of mathematics and science counterintuitive concepts (Brookman-Byrne et al., 2018; Vosniadou, Pnevmatikos, Makris, Lepenioti, et al., 2018).

### Conceptual Change and the Role of Inhibitory Control

Research suggests that mathematics and science learning do not simply involve acquiring new information, rather, existing conceptual frameworks need to be changed and new concepts constructed alongside them (Carey, 2000, 2009; Vosniadou, Pnevmatikos & Makris, 2018, Zaitchik et al., 2014). Children come to the classroom with immature beliefs and theories based on their earlier learning and first-hand experiences of the world (Piaget, 1974). Some new concepts seem counterintuitive as they contradict these naïve theories. For example, we experience the Earth as flat; the ground beneath us looks flat and when a child kicks a football across a pitch, the ball behaves as if it were on a flat surface. These first-hand experiences conflict with the conceptual understanding that the Earth is a spherical body, as taught in primary school science (Allen, 2014). Similarly, in mathematics, early learning that positive numbers increase in magnitude (1 < 2 < 3) can interfere with an understanding that the same integers as negative numbers decrease in magnitude (− 1 > − 2 > − 3) (Bofferding, 2019; Hansen et al., 2017). Perceptual cues can also conflict with learning new concepts. For example, children may naïvely assume that a larger object is always heavier than a smaller object (Nayfeld et al., 2013) or that a 2D shape with a larger surface area is always the shape with the greater perimeter (Babai et al., 2015; Rousselle et al., 2004). These naïve theories and misleading perceptual cues can develop into persistent misconceptions and continue to interfere with reasoning into adolescence and adulthood, despite years of education (McNeil & Alibali, 2005; Verkade et al., 2017; Vosniadou, Pnevmatikos & Makris, 2018).

Evidence suggests that IC is required to prevent prior knowledge, intuitive theories, and misleading perceptual cues from interfering with learning counterintuitive concepts (see Mareschal, 2016). Behavioural studies have demonstrated that solving counterintuitive mathematics problems requires one to inhibit an incorrect strategy or a dominant (i.e. ‘prepotent’) response (Borst et al., 2013; Linzarini et al., 2015; Lubin et al., 2013) and that children and adolescents with better IC perform better on counterintuitive mathematics and science problems (Baker et al., 2011; Brookman-Byrne et al., 2018; Vosniadou, Pnevmatikos, Makris et al., 2018; Zaitchik et al., 2014). Neuroimaging studies have demonstrated activation in prefrontal brain regions (in particular the inferior frontal cortex, dorsolateral prefrontal cortex, and anterior cingulate cortex) during mathematics and science reasoning, which may reflect IC (Babai et al., 2015; Brault Foisy et al., 2015; Fugelsang & Dunbar, 2005; Stavy & Babai, 2010). These brain areas have also been found to be activated more when experts, compared to novices, solve counterintuitive problems, suggesting that experts are able to inhibit intuitive responses (Masson et al., 2014). Learning new mathematics and science concepts may therefore be constrained by the child’s IC abilities. This has implications for how mathematics and science are taught in the classroom and the design of interventions that aim to improve academic achievement (Babai et al., 2015; Mareschal, 2016).

### Cognitive Training and ‘Real-World’ Transfer

The longstanding evidence demonstrating the importance of EF skills to education (Zelazo et al., 2016), alongside evidence that EFs continue developing through childhood and adolescence (Best & Miller, 2010; Crone & Steinbeis, 2017), suggests that cognitive interventions focused on training EFs may be a useful method of improving academic achievement. Evaluations of programmes designed to improve EFs suggest that these skills are trainable (for reviews, see Diamond 2012; Diamond & Lee, 2011; Diamond & Ling, 2016; Jacob & Parkinson, 2015; Serpell & Esposito, 2016). Therefore, an intervention that targets IC could improve children’s learning of new mathematics and science concepts and, in turn, academic achievement. However, these reviews also highlight that training on generic EF tasks (e.g., a go/no-go task, in which participants are required to respond to a certain stimulus and withhold their response to a different stimulus; Trommer et al., 1988), rarely transfer to non-trained tasks. In particular, evidence typically does not demonstrate successful transfer effects following IC training (Spierer et al., 2013; Thorell et al., 2009). For example, Thorell and colleagues found that preschoolers undergoing 5 weeks of working memory training showed significant improvements in trained and untrained working memory and attention tasks relative to controls, but children undergoing equivalent IC training only showed improvement on trained tasks.

This lack of transfer from EF training may reflect the domain-specific ways in which information is processed in the brain. Information processing approaches to cognition represent EF processes as encapsulated modules (e.g., attention module, working memory module, IC module) which can manipulate any type of information, whether it involves for example, the magnitude of numbers or syntactic rules of English grammar (e.g. the ‘central executive’ model of working memory; Baddeley & Hitch, 1994). However, research that has attempted to implement EF processes within neural networks shows that these cognitive control processes are actually embedded within particular domains of knowledge (McClelland & Rogers, 2003; O’Reilly et al., 2010). In this vein, consideration of the neurocomputational basis of cognitive control suggests that IC may be applied to content-specific representations by specific connections, and that part of the training effect is to strengthen these content-specific connections (Botvinick & Cohen, 2014). Therefore, training domain-general skills (such as working memory capacity or general IC) may not have as much impact on the control of knowledge as training these EF skills within a target domain (such as mathematics and science education). Furthermore, EF training which relies on laboratory-based tasks removed from the ‘real-world’ are less likely to be effective than those which train within the context in which they are to be applied (Bryck & Fisher, 2012; Jaroslawska et al., 2016; Moreau & Conway, 2014). There is some evidence that EF training delivered by teachers in the classroom can have positive effects on children’s academic success, at least in the case of working memory training on end of year mathematics and English achievement (Holmes & Gathercole, 2014).

It therefore follows that it may be necessary to embed IC training within the content of the domain to improve mathematics and science counterintuitive reasoning and academic achievement. That is, the intervention would train children to use their IC in the classroom while reasoning about mathematics and science problems from the school curriculum (Mareschal, 2016; Vosniadou, Pnevmatikos & Makris, 2018).

### Current Study

The aim was to (1) demonstrate the presence of misconceptions in primary school children and examine cross-sectional associations between IC, counterintuitive reasoning, and mathematics and science achievement, and (2) evaluate a neurobiologically-informed intervention designed to improve mathematics and science learning. This novel computerised learning activity, called Stop & Think, was designed to embed IC training within mathematics and science content based on the National Curriculum in England (Department for Education, 2013a, 2013b), and be delivered in the classroom during mathematics and science lessons. The intervention was informed by research from cognitive neuroscience on IC, conceptual change, and mathematics and science learning (see Mareschal, 2016; Vosniadou, Pnevmatikos & Makris, 2018). Best practices for EF training (e.g. task novelty, repeated practice, and increasing challenge) reported in reviews of the cognitive training literature, were also carefully considered (Bryck & Fisher, 2012; Diamond & Ling, 2016; Green et al., 2018). Research in technology-enhanced learning, including work in the area of digital game-based learning (Holmes, 2017; Howard-Jones et al., 2014; Howard-Jones et al., 2016) and intelligent learning environments (Grawmayer et al., 2015; Mavrikis et al., 2013; Bernardini et al., 2014; Porayska-Pomsta et al., 2018; Porayska-Pomsta et al., 2013), allowed us to optimise the computer platform to support learning.

We hypothesised that (1) children’s IC would be positively associated with performance on a novel test of mathematics and science counterintuitive reasoning and standardised assessments of mathematics and science academic achievement, and (2) children participating in the Stop & Think intervention would show improved performance on the counterintuitive reasoning test (near transfer) and mathematics and science academic achievement (far transfer) compared to their baseline performance and compared to a control group who underwent teaching as usual.

We were also interested in the mechanism by which any improvements in counterintuitive reasoning may be found and therefore included a more general measure of IC (i.e. a Stroop-like task). However, as the intervention was designed to train children to use their IC within the content-specific domain of mathematics and science, rather than more general IC training, predictions were not made as to whether the intervention would have any benefit to performance on this IC paradigm.

As an investigation of the feasibility of this intervention in a real-world setting, we examined two different modes of delivery within the classroom; a whole-class teacher-led intervention and an individual pupil-led version. As there are documented benefits to both whole-class teaching, allowing teacher guidance and collaborative learning, and independent learning, allowing individualised instruction and pacing (Diamond & Lee, 2011; Wood & O’Malley, 2007), no predictions were made regarding the relative benefits.

Finally, school socioeconomic characteristics (SES) were examined to account for previous research demonstrating that children from low SES neighbourhoods have poorer EFs, academic achievement, and school engagement (Berkowitz et al., 2017; Furlong & Christenson, 2008; Janosz et al., 2000; Lawson et al., 2018). As it is not known whether children from lower SES neighbourhoods have more scope for improvement (due to lower academic and EF baselines), or have less scope for improvement (due perhaps to poorer engagement), no predictions were made regarding the effect of SES on intervention outcomes.

## Method

This project received approval from the Birkbeck Research Ethics Committee.

### Participants

Nine primary schools in London were recruited for the cross-sectional study. Two age groups, 7- to 8-year-olds (Year 3) and 9- to 10-year olds (Year 5), were chosen to allow an investigation across a range of primary school ages, while avoiding Year groups in which minimal science content had yet been taught (i.e. younger Year groups) or in which national testing was taking place (i.e. Year 6). As the intervention was designed as an educational tool for teachers to use in the ‘real-world’ classroom with all pupils in their class, children were not excluded due to disabilities, special educational needs, or any other criteria. Percentages of children with free school meals (FSMs) were taken from the Department for Education (2018) records. The SES profile of the schools varied considerably with the proportion of children with FSM ranging from 3.6 to 40.3% across schools (*M* = 18.9; *SD* = 12.8).

Parents were sent information sheets about the intervention and assessments and given the option to opt-out. Parents of three children opted-out. This yielded a sample of 627 children aged 7.20 to 10.18 years (*M* = 8.53, *SD* = 0.96), with 373 from Year 3 (7.20 to 8.63 years; *M* = 7.78; *SD* = 0.36) and 254 from Year 5 (9.14 to 10.18 years; *M* = 9.64; *SD* = 0.26). Six of these schools (456 children, 267 from Year 3 and 189 from Year 5) also participated in an evaluation of the Stop & Think intervention. For practical reasons, conditions could not be fully randomised. The Stop & Think pupil-led (STP) condition was assigned to classes (either Year 3 or Year 5) with facilities for each child in the class to play Stop & Think on individual computers. Schools without these facilities were assigned to the whole-class Stop & Think teacher-led (STT) condition, with the same spread of classes from each Year assigned to the intervention as in the STP condition where possible. While one Year group in each school participated in either the STP or STT intervention, the other Year group (i.e. Year 3 or Year 5) in each school was assigned to teaching as usual (TAU). This ensured that there were no control-only schools to encourage school participation. One school originally assigned to the teacher-led condition was unable to deliver the intervention due to difficulties with their IT facilities, but agreed to remain in the study as a TAU-only school. Group sizes were as follows: STP, *N* = 102 (Year 3, *n* = 55; Year 5, *n* = 47); STT, *N* = 70 (Year 3, *n* = 24; Year 5, *n* = 46); and TAU, *N* = 284 (Year 3, *n* = 188; Year 5, *n* = 96). Age, gender and SES profiles of children participating in the intervention analyses are reported in Table 1.

**Table 1:** Demographics for children participating in the intervention evaluation (*N* = 456) split by Year group and condition

	Year 3			Year 5		
	STP	STT	TAU	STP	STT	TAU
*N*	55	24	188	47	46	96
Age, *M* (*SD*)	8.07 (0.28)	7.64 (0.34)	7.72 (0.29)	9.65 (0.23)	9.60 (0.20)	9.65 (0.30)
Gender (% male)	40.0	58.3	44.9	57.4	54.5	46.8
School % FSM, *M* (*SD*)	7.1 (−)^a^	3.6 (−)^a^	24.9 (6.2)	21.3 (−)^a^	35.1 (5.9)	16.5 (7.1)

^a^Only one school participated in each of these conditions; therefore, all pupils have the same school free school meals (FSM) data and *SDs* are not reported

### Tasks

#### Mathematics and Science Counterintuitive Reasoning

A 20-item assessment with 10 mathematics and 10 science questions based on content from the National Curriculum for England (Department for Education, 2013a, 2013b) was developed as a pre- and post-intervention measure of counterintuitive reasoning. Unlike most previous studies which have focused on a single misconception, this assessment was designed to cover a broad range of concepts from across the age-relevant curriculum (e.g. decimals, fractions, geometry, living organisms, forces, electricity) to increase the relevance of our findings to primary education. Eight items were based on concepts included in the Stop & Think intervention (although they used different stimuli, question phrasing, and mode of response), and eight items were concepts not included in Stop & Think. Each item consisted of a written question, an image (which was either essential for the question or supported the written text to keep the task engaging), and four multiple-choice response options (see examples in Fig. S1). One response option was correct and three incorrect, with one of the incorrect options being the expected misconception based on the literature. Four additional items were based on mathematics and science concepts that are not commonly associated with a misconception. These four items were not used in the analyses but were included to help prevent children from thinking that there was always a misconception or a ‘trick’ answer (i.e. overall counterintuitive reasoning scores used in analyses refer to the 16 misconception items only). This format was used to develop separate counterintuitive reasoning assessments for the two Year groups, with content of each based on the age-appropriate National Curriculum. Children completed the same assessment at Time 1 (T1) and Time 2 (T2). Items with no response were scored as 0 to reflect an incorrect response.

#### Mathematics and Science Academic Achievement

As a standardised assessment of academic achievement, we used the paper versions of the Progress Test in Mathematics 7 and Progress Test in Science 8 (Year 3) and the Progress Test in Mathematics 9 and Progress Test in Science 9 (Year 5) (GL Assessment, [Bibr CR44][Bibr CR45][Bibr CR46][Bibr CR47]). The Progress Tests assess understanding and application of mathematics and science content from the National Curriculum in England. To reduce the length of the assessment and practice effects, each test was split into two booklets (‘A’ and ‘B’). Children were given one booklet at T1 and the other at T2, with the order of booklet A and B counterbalanced across different classes. Items with no response were scored as 0 to reflect an incorrect response. As children completed different booklets at time 1 and 2, Z-scores were used to compare time 2 to time 1 performance. Z-scores were calculated according to the distribution of time 1 performance for each booklet within each Year group. The same time 1 distribution was then used to calculate Z-scores for the appropriate booklet at time 2 (e.g. the distribution of booklet A scores for Year 3 children completing this booklet at time 1 was used to calculate Z-scores for the Year 3 children completing booklet A at time 2).

#### Inhibitory Control

A pencil-and-paper adaptation of Wright, Waterman, Prescott and Murdoch-Eaton’s (2003) Stroop-like measure of IC for children was used. The pencil-and-paper version allowed us to carry out whole-class assessments in schools that did not have individual child computer facilities available. All children carried out the same pencil-and-paper version for consistency. In a typical Stroop task, conflicting information is presented simultaneously (e.g. the word ‘red’ written with blue ink) and success depends on the inhibition of the dominant information (text ‘red’) while responding to the less salient information (blue ink) (Stroop, 1935). For this adapted task, children were required to identify the body of a line drawn animal (non-dominant information) while inhibiting the animals’ head (dominant information). Black and white hand-drawn images of four animals (cow, pig, sheep and duck) taken from Wright et al.’s task were presented on four sheets of A4 paper in a 3 × 5 grid of 15 animals (total 60 items) in addition to one sheet of four practice stimuli. Each stimulus was presented with the written name of two animals below it, one correct and one incorrect (the name of one of the other three animals). Children were instructed to tick the name of the animal’s body that they could see. Stimuli were either congruent, in which the animal’s head matched the body, or incongruent, in which the head was substituted with the head of one of the other animals (see examples in Fig. S2). A preferential processing of faces is well documented (Johnson, 1993), and therefore, the incongruent stimuli were designed to elicit a Stroop-like interference by requiring children to inhibit the preferred response of the head to correctly identify the body. Two ‘pure’ lists (two sheets of 15 animals) comprised congruent animals only and were followed by two ‘mixed’ lists (two sheets of 15 animals) made up of 50% congruent and 50% incongruent animals in a fixed random order. Across blocks, 50% of the animal heads were facing to the left and 50% to the right to ensure children could not simply use spatial information to ignore the interfering head (see Macleod, 1991). Pilot testing with a group of 10 primary school children (aged 5–11 years) was undertaken to test whether the instructions were clear and to set a time limit for the task. As a result, 12 s per sheet was set to avoid floor or ceiling effects in either block across Year groups. The same task was used for both Year groups.

To try and ensure the association was derived from children who understood the task, children with low scores (mean score across the two pure sheets of 6 or less) at T1 were omitted (population performance, *N* = 453, min = 0.5, max = 15, mean = 9.3, SD = 2.6), which eliminated 8% of data. To control for confounding factors such as reading and writing speed, processing speed or animal recognition, residual scores were used. We calculated whether an individual performed better (i.e. the number of correct responses) on mixed lists than expected given their performance on pure lists by saving the residuals from a linear regression of the full sample (DV: number correct on mixed sheets; IV: number correct on pure sheets).

### Intervention

A computer-based intervention called ‘Stop & Think’ was developed to address the learning of counterintuitive concepts in 7- to 10-year-olds (Year 3 and Year 5 children). It aimed to improve reasoning about counterintuitive concepts by embedding IC training within the content of the subject domain. An integrative approach to the learning experience was taken by bringing together education, psychology and technology-enhanced learning. Informed by research involving virtual characters as learning peers (Porayska-Pomsta et al., 2018; Porayska-Pomsta et al., 2013), Stop & Think was designed to appear like a television gameshow, in which one animated character named Andy acted as the host, posing mathematics and science questions to the user and to three virtual gameshow contestants (see Fig. S3). The tasks encouraged children to repeatedly practise inhibiting their intuitive response in favour of a delayed and more considered response, i.e. to ‘stop and think’, while solving age-relevant mathematics and science problems based on content from the National Curriculum (England). The intervention was intended to train domain-specific IC in two ways:

i) Stop & Think prompt: The Stop & Think prompt was based on research which demonstrates that children perform better on IC tasks when they are forced to delay responding, allowing time for the prepotent response to dissipate and a more considered response to be formed (Diamond et al., 2002; Simpson & Riggs, 2007). In the Stop & Think gameshow, Andy verbally reminded the user to “stop and think” before posing each question. Once the question was presented, the screen was locked (with the question and stimuli visible) while a Stop & Think logo pulsed at the bottom of the screen for 5 s, compelling the child to withhold their prepotent response and giving them time to think more about the question (see examples in Fig. S3).
ii) Contestants’ reasoning: Three virtual game show contestants were built into the intervention to model the ‘stop and think’ IC skill and to provide examples of mathematics and science reasoning. This was informed by research that demonstrates the benefit of collaborative learning through educational tools such as Think-Pair-Share, Concept Cartoons, and ScotSPRinG (Dabell, Keogh, & Naylor, 2008; Kwok & Lau, 2015; Naylor & Keogh, 2003; Tolmie, 2013). In Stop & Think, the virtual contestants presented their thoughts on each question. One contestant presented the correct line of reasoning, while the other two were incorrect (one holding the misconception and one more generally incorrect). This was randomised across contestants. Children could then consider the contestants’ reasoning, which was either presented before they made their response (to help develop their own reasoning) or after they provide the correct response (to reflect upon why this was the correct answer) (see examples in Fig. S3). The order in which these prompts were delivered was adaptive based on the user’s responses (see Fig. S4).

The mathematics and science tasks were developed by compiling a set of misconceptions documented in the literature that were relevant to the age-appropriate National Curriculum (Allen, 2014; Cockburn & Littler, 2008; Gates, 2002; Hansen et al., 2017; Pine et al., 2001; Ryan & Williams, 2007). For example, the misconception that ¼ is greater than ½ occurs due to a natural number bias from the denominators (i.e. 4 > 2) (Ryan & Williams, 2007) and relates to the teaching of fractions in the mathematics National Curriculum for 8-year-olds (Department for Education, 2013a). Similarly, the misconception that forces always result in movement and therefore a stationary object has no forces acting on it (Allen, 2014) is a misunderstanding of the concept of balanced and opposing forces which is set out in the science National Curriculum for 10-year-olds (Department for Education, 2013b). Questions were reviewed by primary school teachers to check their appropriateness.

Sessions were delivered in a fixed order which progressed from concepts based on the curriculum from the previous academic year to more challenging concepts based on the current academic year curriculum. This allowed children to first practise using the ‘stop and think’ skill with familiar concepts, before moving on to apply this IC skill to more difficult or less familiar concepts.

Each session was split equally between one mathematics concept and one science concept and the order in which they were delivered was randomised (see Fig. S5). For each mathematics or science concept, the user (either the individual child for pupil-led, or the whole class directed by the teacher for teacher-led) was presented with an initial question (‘Exploratory’ subtask) followed by up to five questions based on the same concept (‘Structured Practice’ subtasks) but which took on a different response format and/or increased in difficulty. For each question, the user was required to respond by one of five formats using the computer mouse or keyboard: Enter (typing a number in a response box or an equation), Select (click on one or more correct images or text), Sort (drag and drop images or text into two or more buckets), Order (drag and drop numbers or images in a specified sequence, e.g. smallest to largest or first to last) or Construct (drag and drop images to build pictograms or label diagrams) (see examples in Fig. S6). When all subtasks for the session were complete, or 12 min had passed since logging in, the software automatically ended. The next time the user logged in, the session began with a new Exploratory task regardless of progression in the previous session. This aimed to ensure all classes attempted all 30 topics in each domain (mathematics and science) across the 10-week programme.

The Exploratory subtask allowed the user multiple attempts to correctly respond to the question, with progressively greater levels of support offered each time an incorrect response was given (see Fig. S4). In this way, the intervention took a scaffolded learning approach, in which children received support to build on success (Diamond & Lee, 2011). Structured Practice subtasks provided more opportunities to practise the ‘stop and think’ skill at increasing levels of difficulty, as well as to generalise their understanding of the counterintuitive concept to different questions which used novel stimuli and varied response formats. Repeated practice, in particular practice that is progressively more challenging, and task novelty, have previously been found to improve generalisability and longevity of cognitive training benefits (Diamond & Lee, 2018; Ericsson et al., 2009; Ericsson & Towne, 2010; Klingberg et al., 2005; Moreau & Conway, 2014).

Stop & Think was designed to replace the first 12 min of science or mathematics lessons three times a week for 10 weeks (maximum dose of 360 min), rather than providing additional mathematics and science content to lessons as usual. The intervention could either be used as a whole class, with the teacher leading the session on the classroom interactive whiteboard (teacher-led, STT), or individually with each child interacting with the software on their own school computer wearing headphones (pupil-led, STP). The same software was used for both conditions, but teacher-input differed. In STP, each child moved through the subtasks in their own time within the 12-min session. This allowed optimal individualised pacing and instruction, which has been suggested as best practice for EF training (Diamond & Lee, 2011). Teachers were present in the classroom but instructed to offer support only for any technical issues (e.g. problems logging in or the computer freezing) or re-reading the text on screen if requested, but not to provide help in terms of the mathematics and science content. In STT, teachers were given flexibility to decide how best to use the software as a whole-class activity with suggestions provided, such as asking for a volunteer to offer their answer or taking a class vote. In STT, but not STP, children could discuss the task. However, children needed to sit quietly whenever the Stop & Think logo was pulsing to encourage them to take the time to ‘stop and think’ about the task, ensuring consistent application of the ‘active agent’, i.e. the within-domain IC training. Teachers could re-read the text to the class or re-iterate any instructions or prompts (such as "remember to stop and think"), but again were instructed not to provide help in terms of the mathematics and science content. Teachers were also asked to allow children to make mistakes so that they could take advantage of the levels of support offered in the software when incorrect responses are made and to practise the ‘stop and think’ skill as much as needed.

### Procedure

A researcher visited the schools to install the software and provide training to teachers. The training session lasted approximately 45 min during which the purpose and development of the intervention were discussed, a demonstration of the software was given, and teaching staff had the opportunity to ask questions. An accompanying handbook was given to each member of participating staff. An emphasis was placed on the importance of delivering the intervention for 15 min at the start of a mathematics or science lesson, three times per week.

All pre- and post-intervention assessments were carried out by a researcher, in the classroom with the whole class. The counterintuitive reasoning test was carried out first, followed by the Stroop-like chimeric animals task, then the mathematics and science achievement booklets, with short breaks between tests. Assessments were explained to children as tasks to help scientists find out what children find easy and difficult and to help teachers know how best to support children’s learning. Participants were not told the correct answers or given any indication of their performance, and it was explained that responses were independent of school assessments.

For the counterintuitive reasoning test, each question and four response options were presented on the classroom interactive whiteboard. Children were told that only one response was correct but were not told that a misconception was present. Each question was read out in turn by the researcher, then children were given 20 s to respond by ticking one of the four options on a paper answer sheet. For the IC task, instructions and example items were presented to the whole class on the interactive whiteboard and read out by the researcher. Children were told that sometimes the head and body would match and sometimes they would not, but were not told that there would be ‘pure’ and ‘mixed’ sheets. Examples of two congruent and two incongruent stimuli were presented on the whiteboard and the researcher explained the answers. The question ‘Which animal’s body can you see’ remained on the whiteboard for the duration of the task, was printed at the top of each answer sheet, and was repeated by the researcher before children began each sheet. Children were first asked to complete an untimed practice sheet with four stimuli (two congruent and two incongruent), and the researcher went through the answers with the whole class. For the main task, children were told they had 12 s for each sheet to respond to as many items as they could. Once 12 s had passed on the stopwatch, the researcher told the class to “Stop. Hands on heads” and demonstrated this. The procedure was then repeated for all four sheets. Children completed the same counterintuitive reasoning and Stroop-like tests at T1 and T2. For Year 3 children, questions on the Progress Test in Mathematics 7 were read out to the whole class by the researcher and children marked their response in a booklet. The other Progress Tests were designed to be read by the children themselves. For these, two example items were completed as a whole class and then children were given 30 min to complete each booklet individually.

The 10-week intervention began in schools 2–4 weeks after the T1 assessments, determined by each schools’ capabilities to begin (i.e. school holidays, teacher absence, or school IT facilities). Sessions started and completed were automatically logged online through a remote server. The lead author also visited each school mid-way through the intervention period to ask teachers whether they were running the sessions three times a week, which lesson they were running sessions in, and to answer any questions regarding the implementation of the intervention. T2 assessments were carried out 1- 2 weeks after the intervention had finished.

### Data Analysis

Some participants did not complete all assessments due to pupil absence at the time of testing or schools opting out of some assessments. In particular, achievement data were not available for Year 3 STP or Year 5 STT as some schools preferred not to include these assessments due to time constraints and the demand on pupils to complete multiple assessments. To optimise on the large *N*s available for some of our assessments (i.e. the T1 counterintuitive reasoning and Stroop tasks), all data available for each analysis were used. Therefore, participant numbers varied by analyses and *N*s are reported with the results of each analysis. Items with no response on the counterintuitive reasoning task and academic achievement assessments were scored as 0 to reflect an incorrect response. The percentage of mathematics and science items with no response at T1 and T2 for each Year group are presented in the supplementary materials (Table S1).

#### Cross-Sectional Analyses

T1 performance on the counterintuitive reasoning test was analysed to assess whether children held mathematics and science misconceptions. Paired samples *t* tests were used to compare the number of misconception errors (i.e. the incorrect response option based on a misconception documented in the literature) to other errors (i.e. an incorrect response option not based on a common misconception) for each Year group. Similarly, to test for a baseline Stroop effect, paired samples *t* tests were used to compare mean scores on the pure list to mean scores on the mixed list for each Year group. Higher accuracy on the pure list compared to the mixed list would show a Stroop effect (i.e. poorer accuracy when IC is required).

To identify potential confounds of age or SES, Pearson’s correlations were carried out for age (within Year group) and SES (whole school percentage of children eligible for free school meals) with performance on counterintuitive reasoning, academic achievement, and IC at T1. Pearson’s correlations were also carried out to examine the association between counterintuitive reasoning, academic achievement, and IC at T1 for each Year group.

#### Intervention Effects

The effect of Stop & Think on (a) counterintuitive reasoning, (b) academic achievement and (c) IC were analysed using ANCOVAs, with T2 performance as the dependent variable and T1 performance (mean-centred) on the same measure as a covariate. We assessed whether intervention condition (STT, STP and TAU) modulated the intervention effect for each Year group separately. In line with the exploratory nature of our investigations regarding mode of delivery, separate paired analyses were run first comparing TAU to the intervention conditions combined, and then comparing TAU to STT and STP separately.

## Results

### Cross-Sectional Analyses

**Fig. 1:**
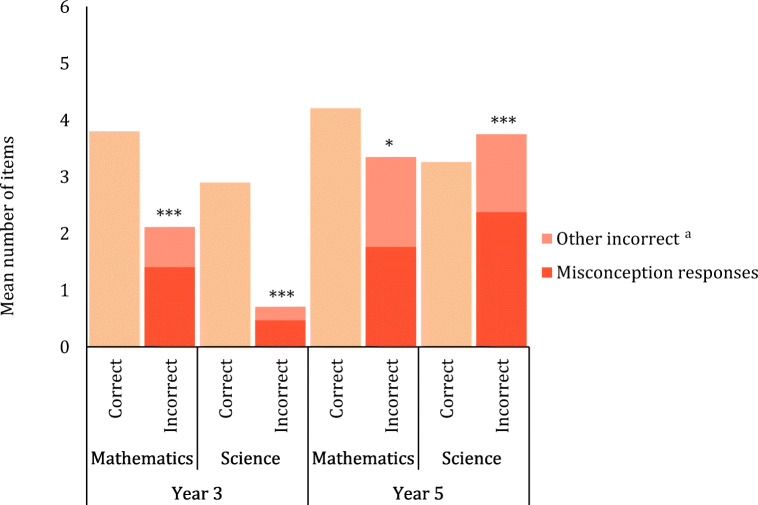
Correct and incorrect responses on the counterintuitive reasoning task for Year 3 (*N* = 351) and Year 5 (*N* = 243) children at T1. Significance levels denote the results of paired samples *t* tests comparing incorrect response types (i.e. misconception responses vs. other incorrect responses for each Year group separately. *p < .05, **p < .01, ***p < .001. ^a^Number of ‘other incorrect’ responses were divided by two to allow a comparison to misconception errors (each item had one misconception response option and two other incorrect response options). Note. Year 3 and Year 5 completed different counterintuitive reasoning tests comprising age-appropriate mathematics and science content

**Fig. 2:**
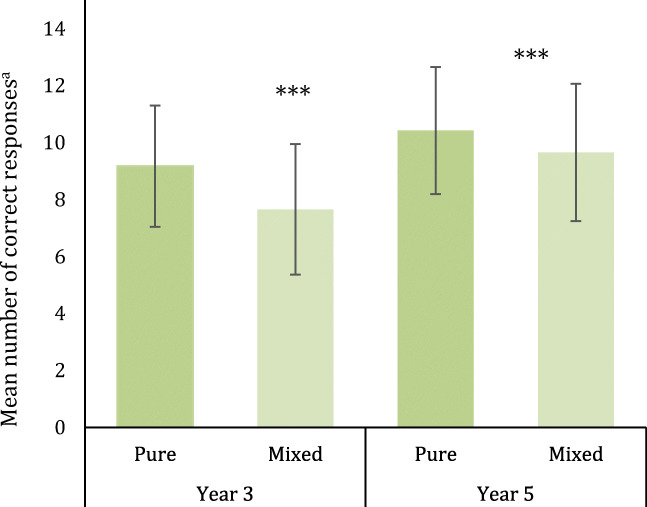
Accuracy on pure and mixed sheets of the Stroop-like inhibitory control task for Year 3 (*N* = 264) and Year 5 (*N* = 187) children at T1. Significance levels denote the results of paired samples *t* tests comparing pure and mixed sheets (i.e. the Stroop inhibitory control effect) for each Year group separately. *p < .05, **p < .01, ***p < .001. ^a^Mean across two pure sheets and two mixed sheets (i.e. maximum possible score = 15)

The number of participants with data for cross-sectional analyses at T1 was *N* = 594 for the counterintuitive reasoning task, *N* = 451 for the IC Stroop task, *N* = 283 for mathematics achievement, and *N* = 129 for science achievement (number of participants per Year group for each analysis are reported in Figs. 1 and 2 and Tables 2 and 3). Achievement data were not available for Year 3 STP or Year 5 STT due to some schools opting out of these assessments. Gender did not significantly modulate any dependent variable at the .05 level and therefore was not considered further.

**Table 2:** Pearson’s correlations at Time 1 for Year 3 children

		Counterintuitive reasoning Overall	Counterintuitive reasoning Mathematics	Counterintuitive reasoning Science	Mathematics achievement	Science achievement ^a^	Stroop-like inhibitory control
Age	*N*	224	224	224	145	67	218
	*r*	.12	.15*	.01	.08	− .17	− .11
	*p*	.073	.022	.943	.333	.182	.103
Free school meals	*N*	350	350	350	146	–	265
	*r*	− .20***	− .20***	− .09	− .33***	–	− .05
	*p*	< .001	< .001	.096	< .001	–	.381
Mathematics achievement	*N*	132	132	132	–	67	145
	*r*	.51***	.54***	.13	–	.52***	.08
	*p*	< .001	< .001	.157	–	< .001	.319
Science achievement	*N*	61	61	61	67	–	66
	*r*	.49***	.53***	.16	.52***	–	.33**
	*p*	< .001	< .001	.221	< .001	–	.006
Stroop-like inhibitory control	*N*	242	242	242	145	66	–
	*r*	.13*	.07	.14*	.08	.33**	–
	*p*	.043	.280	.030	.319	.006	–

^a^ Only one school in each Year group completed the Science achievement test; therefore, correlations with school SES were not conducted

*p < .05; **p < .01; ***p < .001

**Table 3:** Pearson’s correlations at Time 1 for Year 5 children

		Counterintuitive reasoning	Counterintuitive reasoning Mathematics	Counterintuitive reasoning Science	Mathematics achievement	Science achievement^a^	Stroop-like inhibitory control
Age	*N*	208	208	208	135	62	174
	*r*	.07	.07	.04	.05	.06	.10
	*p*	.349	.334	.577	.555	.666	.210
Free school meals	*N*	243	243	243	137	–	188
	*r*	− .22***	− .26***	− .09	− .31***	–	− .11
	*p*	< .001	< .001	.151	< .001	–	.118
Mathematics achievement	*N*	133	133	133	–	62	136
	*r*	.51*	.61*	.16	–	.50***	.39***
	*p*	< .001	< .001	.076	–	< .001	< .001
Science achievement	*N*	64	64	64	62	–	63
	*r*	.53***	.46***	.41***	.50***	–	.17
	*p*	< .001	< .001	< .001	< .001	–	.181
Stroop-like inhibitory control	*N*	177	177	177	136	63	–
	*r*	.30***	.29***	.19*	.39***	.17	–
	*p*	< .001	< .001	.011	< .001	.181	–

*p < .05; **p < .01; ***p < .001

^a^Only one school in each Year group completed the Science achievement test;, therefore, correlations with school SES were not conducted

### Baseline Performance on Counterintuitive Reasoning and Inhibitory Control

Paired samples *t* tests were used to compare the type of errors children made on the counterintuitive reasoning test at T1 (Fig. 1). Year 3 children made significantly more misconception errors than other errors for mathematics items, *t*_(350)_ = 20.76, *p* < .001, Cohen’s *d* = 1.11, and for science items, *t*_(350)_ = 12.89, *p* < .001, Cohen’s *d* = 0.69. Year 5 children made more misconception errors than other errors for mathematics items *t*_(242)_ = 2.38, *p* = .018, Cohen’s *d* = 0.15, and for science items, *t*_(242)_ = 11.02, *p* < .001, Cohen’s *d* = 0.71 (Fig. 1).

To assess whether a Stroop effect was present at baseline, paired samples *t* tests were used to compare mean scores on pure sheets compared to mean scores on mixed sheets at T1 (Fig. 2). There was a significantly higher mean number of correct trials on pure sheets compared to mixed sheets (i.e. a Stroop effect) in Year 3, *t*_(263)_ = 7.74, *p* < .001, Cohen’s *d* = 0.48, and Year 5, *t*_(186)_ = 5.05, *p* < .001, Cohen’s *d* = 0.37.

### Correlations Between Free School Meals and Children’s Cognitive Performance

Pearson’s correlation analyses are presented in Tables 2 (Year 3) and 3 (Year 5). Higher school percentage of FSM was significantly associated with lower overall counterintuitive reasoning scores for Year 3 and Year 5 (and with mathematics counterintuitive items, but not science counterintuitive items for each Year group). Higher percentage of FSM was also significantly associated with poorer mathematics achievement for Year 3 and Year 5. As only one school in each Year completed the science achievement test, correlations with FSM were not conducted. FSM was not significantly associated with IC for Year 3 or Year 5. As FSM data were only available on a school level, SES was not included in the within-subject intervention effect analyses. Nevertheless, these results suggest that SES may confound some of the associations and therefore must be considered when interpreting results.

### Correlations Between Counterintuitive Reasoning and Academic Achievement

Counterintuitive reasoning performance (overall scores and mathematics items alone) was significantly associated with mathematics and science academic achievement for children in Year 3 (Table 2) and Year 5 (Table 3). However, performance on science counterintuitive items alone was not significantly associated with mathematics or science achievement for Year 3 children, or with mathematics achievement for Year 5 children.

### Correlations Between Inhibitory Control and Counterintuitive Reasoning and Academic Achievement

For Year 3 children, IC was significantly associated with science achievement, but not mathematics achievement, and with overall counterintuitive reasoning performance (and science counterintuitive items, but not mathematics counterintuitive items) (Table 2). For Year 5 children, IC was significantly associated with mathematics achievement, but not science achievement, and with overall counterintuitive reasoning performance (and mathematics and science counterintuitive items separately) (Table 3).

Within Year groups, age was not significantly associated with IC, overall counterintuitive reasoning performance, or academic achievement, and was therefore not included in further analyses.

### Intervention Effects

Accurate data on the number of sessions completed was not available as some sessions ran offline due to school IT issues. However, on mid-intervention visits to the schools, most teachers reported that they had been running sessions three times per week at the start of mathematics lessons. Some teachers did not manage to run three sessions every week but ‘caught-up’ by running more sessions the following week.

The number of participants with data available for intervention effects varied by analysis and are reported in Table 4. Intervention condition effects were Bonferroni corrected for multiple comparisons (Table 4).

**Table 4:** Summary of intervention condition effects by Year group

		Year 3			Year 5		
		Intervention (STT and STP) vs. TAU	STT vs. TAU	STP vs. TAU	Intervention (STT and STP) vs. TAU	STT vs. TAU	STP vs. TAU
Counterintuitive reasoning	*N* η_p_^2^	233 .062***	180 .067***	212 .041**	172 n.s.	128 n.s.	134 n.s
Science achievement^a^	*N* η_p_^2^	–	67 .077*	–	–	–	64 n.s.
Mathematics achievement^a^	*N* η_p_^2^	–	144 n.s.	–	–	–	127 .038^b^
Stroop-like inhibitory control	*N* η_p_^2^	262 n.s.	208 n.s.	238 n.s.	187 n.s.	140 n.s.	142 n.s.

*STT* Stop & Think, teacher-led, *STP* Stop & Think, pupil-led, *TAU* teaching as usual

*p < .05; **p < .01; ***p < .001 (after Bonferroni correction)

^a^Academic achievement data was not available for Year 3 pupil-led or Year 5 teacher-led

^b^*p* = 029 but was not significant following the Bonferroni correction

#### The Effect of Intervention Condition on Counterintuitive Reasoning

**Fig. 3:**
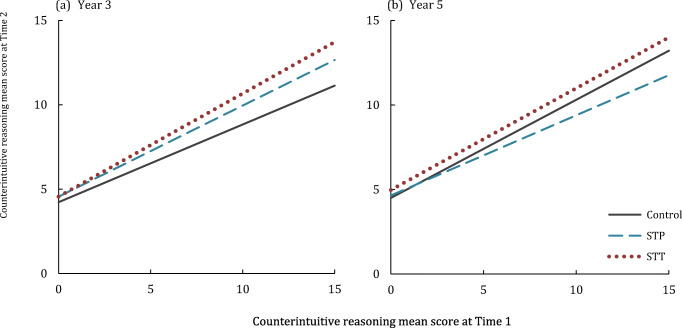
Intervention effect on counterintuitive reasoning test performance for teacher-led (STT) and pupil-led (STP) Stop & Think interventions compared to teaching as usual (Control), for **a** Year 3 children and **b** Year 5 children

In the first comparison, intervention conditions (STT, STP) were combined and compared against TAU. There was a significant intervention effect compared to TAU on counterintuitive reasoning performance for Year 3 [*F*_(1,230)_ = 15.2, *p* < .001, η_p_^2^ = .062], but no significant intervention effect for Year 5 children [*F < 1*]. Next STT and STP were compared to TAU individually (Fig. 3). There was a significant STT intervention effect on counterintuitive reasoning performance in Year 3 [*F*_(1,177)_ = 12.76, *p* < .001, η_p_^2^ = .067], but not in Year 5 [*F*_(1,125)_ = 2.54, *p* = .114, η_p_^2^ = .020]. Similarly, there was a significant STP intervention effect on counterintuitive reasoning performance in Year 3 [*F*_(1,209)_ = 8.95, *p* < .01, η_p_^2^ = .041]. The STP intervention effect on counterintuitive reasoning performance in Year 5 did not survive a Bonferroni correction [*F*_(1,134)_ = 4.63, *p =* .033, η_p_^2^ = .034]. Follow-up analyses to compare STT to STP for Year 3 children showed improvements in counterintuitive reasoning performance over time were significantly larger for STT [*F*_(1,71)_ = 1.34, *p* = .252, η_p_^2^ = .018].

#### The Effect of Intervention Condition on Academic Achievement

**Fig. 4:**
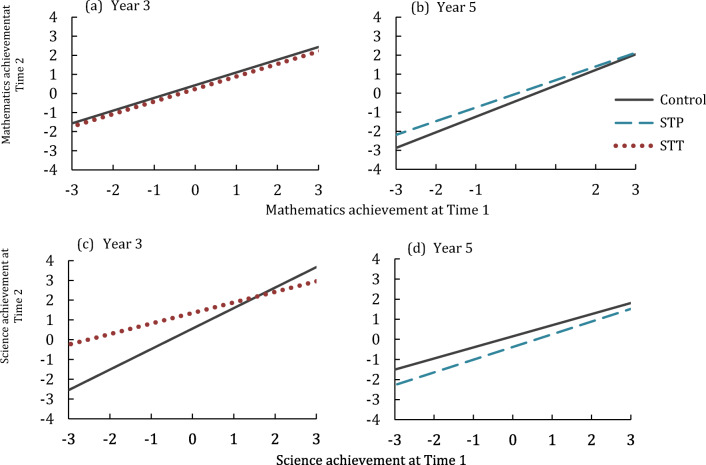
Intervention effect on mathematics achievement for **a** Year 3 children in the teacher-led Stop & Think intervention (STT) compared to teaching as usual (control) and **b** Year 5 children in the pupil-led Stop & Think intervention (STP) compared to teaching as usual (control). Intervention effect on science achievement for **c** Year 3 children in the teacher-led Stop & Think intervention (STT) compared to teaching as usual (control) and **d** Year 5 children in the pupil-led Stop & Think intervention (STP) compared to teaching as usual (control). Data are Z-scores computed on T1 performance

Academic achievement data was not available for Year 3 STP or Year 5 STT due to some schools opting out of these assessments. For Year 3 children, STT was compared to TAU. There was a significant intervention effect on science achievement [*F*_(1,64)_ = 5.37, *p* < .05, η_p_^2^ = .077], but not on mathematics achievement [*F*_(1,141)_ = 1.36, *p* = .246 η_p_^2^ = .010]. For Year 5 children, STP was compared to TAU. The effect of intervention on mathematics achievement did not survive the Bonferroni correction [*F*_(1,124)_ = 4.85, *p =* .029, η_p_^2^ = .038], and there was no significant effect on science achievement [*F*_(1,61)_ = 1.94, *p* = .169, η_p_^2^ = .031] (Fig. 4).

#### Effect of Intervention Condition on Inhibitory Control

**Fig. 5:**
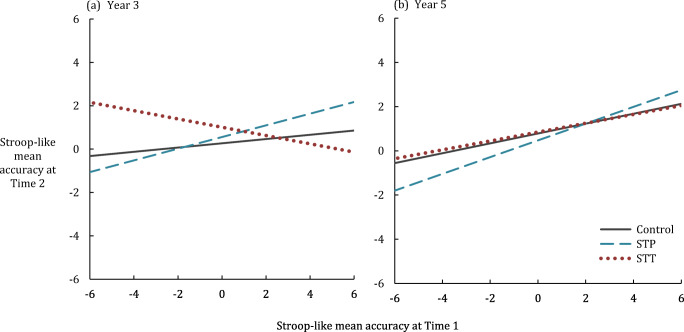
Intervention effect on Stroop-like inhibitory control for teacher-led (STT) and pupil-led (STP) Stop & Think interventions compared to teaching as usual (control), for **a** Year 3 children and **b** Year 5 children. Data are residual accuracy scores derived from a calculation of whether the individual’s performance on mixed sheets was better than expected given their performance on pure lists, against a linear regression from the full sample

When intervention conditions (STT, STP) were combined and compared against TAU, there was no significant intervention effect on the Stroop-like IC measure for Year 3 [*F*_(1,259)_ = 2.18, *p* = .139, η_p_^2^ = .008] or Year 5 [*F* < 1]. When STT and STP were compared to TAU individually, a significant effect was observed in Year 3 STT only, but this did not survive a Bonferroni correction [STT Year 3: *F*_(1,205)_ = 3.82, *p* = .05, η_p_^2^ = .023; STT Year 5: *F* < 1; STP Year 3: *F* < 1; STP Year 5: *F* < 1] (Fig. 5).

## Discussion

The current study examined mathematics and science counterintuitive reasoning in 7- to 10-year-old children and the association with academic achievement and IC. First, we confirmed that children did hold misconceptions in both mathematics and science based on content from the National Curriculum, as we found that children were more likely to respond to counterintuitive reasoning questions with a misconception response than a more general incorrect response, with large effect sizes (small effect size for Year 5 mathematics). We then explored the idea that IC skills may contribute to children’s counterintuitive reasoning and mathematics and science academic achievement. We found that greater IC on a Stroop-like task was associated with better performance on a counterintuitive reasoning task and greater mathematics and science academic achievement scores, although the associations varied between the Year groups, with stronger associations with science in Year 3, and with mathematics in Year 5. These findings support the idea that children with more proficient IC are better at reasoning about counterintuitive problems, perhaps due to the ability to withhold an intuitive response in favour of a more considered response (Mareschal, 2016). While only cross-sectional, these findings are in-line with recent evidence of an association between IC and performance on tests involving a range of mathematics and science misconceptions (Brookman-Byrne et al., 2018; Vosniadou, Pnevmatikos, Makris, Lepenioti, et al., 2018). These results also extend previous research by examining associations with academic achievement which increases the relevance of these findings for education.

Next, we evaluated a new classroom-based computerised intervention, Stop & Think, designed to train IC embedded within the context of the learning domain, i.e. practising withholding pre-potent responses to counterintuitive concepts based on the mathematics and science National Curriculum in England. We evaluated two intervention conditions (Teacher-led, STT and Pupil-led, STP) for two school Year groups (Year 3 and Year 5) and predicted transfer effects from the intervention to counterintuitive reasoning task performance (near transfer) and mathematics and science achievement (far transfer). The intervention showed some promising near and far transfer effects, but results varied by mode of intervention delivery and Year group. Results indicated near transfer for Year 3 children (but not Year 5) from the intervention to counterintuitive reasoning performance in both STT and STP with small to moderate effect sizes. Furthermore, for Year 3 children (but not Year 5), there was evidence of transfer to improved performance on novel concepts not seen in the Stop & Think intervention (see Supplementary Materials), suggesting that children were applying the ‘stop and think’ skill to new counterintuitive concepts, rather than simply recalling the correct answers to the concepts practised in the intervention. There was also a moderate effect for far transfer of STT in Year 3 to science achievement (not mathematics), but no significant transfer of STP in Year 5 to mathematics or science achievement. However, for practical reasons, conditions could not be fully randomised and data was not available for Year 3 STP or Year 5 STT, limiting the conclusions that can be made regarding far transfer.

Overall, the results provide preliminary evidence of an intervention with potential transferable benefits to children’s academic achievement, at least in terms of Year 3 science. Previous studies report that training on laboratory-type EF tasks benefit performance on similar EF tasks, but evidence of transfer to improvements in ‘real-world’ abilities is lacking (Diamond & Ling, 2016; Jacob & Parkinson, 2015; Kray & Ferdinand, 2013; Serpell & Esposito, 2016). Similarly, educational programmes are often designed to target a specific misconception (e.g. understanding rational numbers in light of a natural numbers bias; Vamvakoussi et al., 2018), which require considerable time and resources from the teacher. In contrast, this was the first evaluation of an intervention informed by neuroscience that aimed to improve counterintuitive reasoning through IC training embedded within age-appropriate content from the mathematics and science curricula. Unlike many EF training programs which utilise laboratory-type cognitive tasks, we based all mathematics and science content on the National Curriculum, with tasks validated by teachers and delivered within mathematics and science school lessons (i.e. domain-specific training). Rather than delivering mathematics and science content teaching or focusing on explaining a specific misconception, this intervention used examples of counterintuitive reasoning to train children when and how to use IC (i.e. to ‘stop and think’), which they could then apply to learning more broadly. The current findings provide some support for this embedded domain-specific training approach.

### Inhibitory Control

As predicted, there was a positive association between IC and counterintuitive reasoning performance (science items only for Year 3 pupils and both mathematics and science items for Year 5 pupils) and both science achievement (for Year 3 pupils) and mathematics achievement (for Year 5 pupils) at baseline. However, due to the cross-sectional nature of these analyses, we do not know whether a common causal factor, such as IQ or reading ability, family background, or teaching, was driving performance on both the counterintuitive reasoning test and academic achievement. Further research is needed which controls for these potential confounds to better understand the associations found here.

Predictions were not made regarding transfer effects from the intervention to Stroop-like IC performance, due to the artificial laboratory-type nature of this task. While it was interesting to examine whether the benefits of Stop & Think training transferred to performance on a traditional IC paradigm, the aim of this intervention was not to train general IC, but rather to train children to use this skill in the context of mathematics and science counterintuitive concepts. Nevertheless, the lack of transfer to IC may be due to the limitations of the measure used. EFs are notoriously difficult to measure, with considerable overlap between different EF skills and questionable ecological validity of laboratory-type cognitive tasks (Chaytor et al., 2006; Green et al., 2018; Zelazo et al., 2016). Further, there are thought to be many different types of IC (Nigg, 2000). It may be that the IC skills required for mathematics and science reasoning are not best measured by this Stroop-like task. For example, Cragg and colleagues (2017) found that performance on a numerical IC task, but not an animal-size Stroop-like task, predicted individual differences in factual knowledge and procedural skills in mathematics in children and adults. Moreover, while adapted from previous research which examined its suitability for use with children (Wright et al., 2003), our pencil-and-paper version of the Stroop-like chimeric animals task (designed for practical reasons to test whole-classes without requiring school computer facilities), may not be a sufficiently sensitive measure of IC. This needs further investigation with alternative measures to examine whether the intervention improves the learning of counterintuitive problems through an improved ability to withhold a pre-potent response in favour of a more considered response. Finding reliable outcome measures that reflect real-world EF performance in the evaluation of cognitive training remains a challenge (Green et al., 2018).

### Mode of Intervention Delivery

As this intervention was developed to offer benefit within the classroom, the mode of delivery (i.e. Teacher-led or Pupil-led) was examined. Both Year groups combined benefitted more from the Teacher-led intervention in terms of counterintuitive reasoning performance, and Year 3 children also benefitted from Teacher-led delivery in transfer to science achievement. However these results need to be interpreted with caution as achievement data was missing for Year 3 STP and Year 5 STT. Nevertheless, the Pupil-led intervention also showed promise for Year 3 children with transfer effects to counterintuitive reasoning performance.

While teachers were instructed not to provide help with the mathematics or science content in either condition, it may be that the teacher’s involvement in the Teacher-led condition helped children to stay on task and motivate them to succeed (Smith et al., 2017). Moreover, children may have gained additional benefits from the teacher interacting with the software themselves, as this may have increased teacher investment in the training and possibly lead to the teacher implementing the ‘stop and think’ skill in other lessons. Previous research has found that EF training delivered as add-on sessions to the school curriculum are less effective than when EF skills are supported and appropriately challenged throughout the school day (Bodrova & Leong, 2006). Working as a group in the Teacher-led condition likely also promoted peer discussion, which has previously been found to improve children’s engagement and learning (Chun-Lok Fung et al., 2016; Howe et al., 2007; Thurston et al., 2010; Tolmie et al., 2010; Wood & O’Malley, 2007). For example, Tolmie et al. (2010) found that 5–8-year olds’ learning about road safety progressed the most when both adult guidance and peer discussion were utilised (compared to adult or peer support alone).

While there was no clear optimal mode of delivery (and an incomplete design for transfer to academic achievement), it should be noted that STP was more difficult to deliver in practice given the demands on the schools’ computer resources. Given that the aim was to develop and evaluate an ecologically valid EF training programme embedded within regular school lessons, these practical issues need to be given careful consideration in the development and evaluation of real-world interventions. This initial investigation of how such an intervention is best delivered and the feasibility of implementation within a classroom is an important step forward in the development of school-based EF interventions, which is currently lacking in the cognitive training literature (Green et al., 2018).

### Socioeconomic Status

A subsidiary finding was that SES was associated with counterintuitive reasoning and mathematics achievement (science achievement data was not available for school-level analyses), but not Stoop-like IC. This partially supports previous findings of an association between low SES and poorer academic achievement (Berkowitz et al., 2017; Lawson et al., 2018). In the current study, SES information was only available on a school level. However, the significant associations between FSM with counterintuitive reasoning and mathematics achievement suggest SES must be considered as a potential confound when interpreting these results. For example, in the current study, it may be that low SES was driving the association between counterintuitive reasoning and academic achievement. These findings highlight the need for a larger study that can control for SES, preferably measured on an individual level.

### Future Research

Importantly, the design of this study ensured that intervention benefits could not simply be attributed to additional mathematics and science curricula content, as Stop & Think replaced the first 12 min of regular mathematics or science lessons and therefore these children received the same approximate dosage of mathematics and science content as children in TAU. Nevertheless, while the intervention was designed to train children to use their IC when faced with mathematics and science problems, it may have worked by some other means, such as simply being a novel activity that engaged children in learning, or by children having an expectation of benefits from participating (Bayraktar, 2001; Boot et al., 2013; Diamond & Ling, 2011; Green et al., 2018). Including an active control, in which some children participate in a computer task that does not target IC, would help in our understanding of what lead to the improvements found here. *It would also be worthwhile to measure children’s expectations of taking part. Participant expectations have been found to confound outcomes, yet are largely ignored in intervention research (*Foroughi et al., 2016; Green et al., 2018; Stothart et al., 2014; Tiraboschi et al., 2019).

Future research could also examine the optimal dosage of this type of training (both the duration and frequency of the IC ‘stop and think’ prompt, as well as the duration and frequency of sessions), which will need to balance training exposure with the practicalities of implementing this as part of regular school lessons. Similarly, few studies have examined the long-term effects of EF training and those that have, have often found that *once training ends, the benefits diminish* (e.g. Ball et al., 2002, Klingberg et al., 2005, Willis et al., 2006*).* Longer-term outcomes of the Stop & Think intervention could be explored to account for any sleeper effects, in which real-world application of these skills may be delayed (Green et al., 2018), as well as examining long-term stability of the immediate benefits to counterintuitive reasoning and academic achievement.

Finally, it would be interesting to evaluate this intervention in terms of any structural or functional brain changes. Stop & Think was informed by evidence from neuroscience about the operation of cognitive control (Thomas et al., 2018). In particular, the intervention was grounded in evidence of the involvement of regions supporting IC, such as the prefrontal cortex and anterior cingulate cortex, in mathematics and science reasoning (see Mareschal, 2016). Therefore, the current finding of behavioural improvements could be extended through an examination of any neural changes following intervention to provide convergent evidence of the involvement of the proposed cognitive processes.

### Summary and Implications

In summary, this study found preliminary evidence that participating in the Stop & Think intervention produced both near and far transfer effects, with both Teacher-led and Pupil-led conditions showing promise. While results differed by Year group and mode of delivery, these initial findings of training gains in counterintuitive reasoning and academic achievement suggest that it may be possible to intervene in the learning of counterintuitive concepts through cognitive training delivered by the teacher in the classroom. Addressing counterintuitive reasoning in primary school could prevent persistent misconceptions impeding the learning of more complex concepts in later education (see Verkadeetal et al., 2017). Compared to interventions that focus on literacy skills, there have been few that target mathematics and especially science in primary school. Yet mathematics and science are domains of key economic importance (Hanushek & Woessmann, 2008; Morse, 2018; Rothwell, 2013). Therefore, there could be both educational and economic gains from using this type of IC training as an educational tool within primary school lessons. Future work now needs to investigate whether the benefits found in this study are replicated in a larger-scale randomised controlled trial to establish the value of schools implementing this type of IC training in the real-world classroom.

## Electronic supplementary material

ESM 1 (DOCX 3573 kb)
