# Supplementary material for: Domain-Specific Inhibitory Control Training to Improve Children’s Learning of Counterintuitive Concepts in Mathematics and Science
Source: J Cogn Enhanc. 2019 Dec 12;4(3):296–314. doi: 10.1007/s41465-019-00161-4 (PMC7410229; doi:10.1007/s41465-019-00161-4)
Supplement: Supplementary file 1 — (DOCX 3573 kb) [file 41465_2019_161_MOESM1_ESM.docx]

**Supplementary materials**

*Intervention effects on concepts used in Stop & Think and novel concepts*

The counterintuitive reasoning task comprised both concepts included in the Stop & Think (S&T) intervention (eight ‘S&T-like’ items) and concepts not included in Stop & Think (eight ‘novel’ items). In addition to our primary ANCOVAs on the counterintuitive reasoning task, performance on familiar and novel counterintuitive concepts were analysed separately to assess whether any intervention effects were found on familiar counterintuitive concepts only, or whether there was transfer to novel counterintuitive concepts. The dependent variable was Time 2 performance, and Time 1 performance (mean-centred) on the same measure was included as a covariate. Mathematics and science items were not analysed separately here, as splitting by subject and novelty would reduce the number of items that scores were based on.

For Year 3 children, there was a significant intervention effect (Stop & Think teacher-led; STT and Stop & Think pupil-led; STP combined) compared to TAU on counterintuitive reasoning performance for S&T-like concepts [*F*_(1,230)_ = 19.0, *p* < .001, η_p_^2^ = .076] and novel concepts [*F*_(1,230)_ = 6.74, *p =* .010, η_p_^2^ = .028]. For Year 5 children, there were no significant intervention effects (STT and STP combined compared to TAU) on S&T-like concepts [*F*_(1,169)_ = 2.44, *p* = .120, η_p_^2^ = .014] or novel concepts [*F*_(1,169)_ = 2.51, *p* = .115, η_p_^2^ = .015]. When the intervention conditions were compared to TAU individually (with a critical p-value of .025 following a Bonferroni correction for the two comparisons), there was a significant Year 3 STT intervention effect on S&T-like concepts [*F*_(1, 177)_ = 21.28, *p* < .001, η_p_^2^ = .107], but not on novel concepts [*F*_(1, 177)_ = 1.27, *p* = .261, η_p_^2^ = .007]. However, there was a significant Year 3 STP intervention effect on both S&T-like concepts [*F*_(1, 209)_ = 9.01, *p* = .003, η_p_^2^ = .110] and on novel concepts [*F*_(1, 209)_ = 7.09, *p* = .008, η_p_^2^ = .033]. For Year 5 children there was a significant STT intervention effect for novel concepts [*F*_(1, 125)_ = 5.61, *p* = .019, η_p_^2^ = .043], but not for S&T-like concepts [*F* < 1]. The STP intervention effect on S&T-like concepts in Year 5 did not survive the Bonferroni correction [*F*_(1,134)_ = 4.32, *p* = .040, η_p_^2^ = .032], and there was no significant effect on novel concepts [*F* < 1].


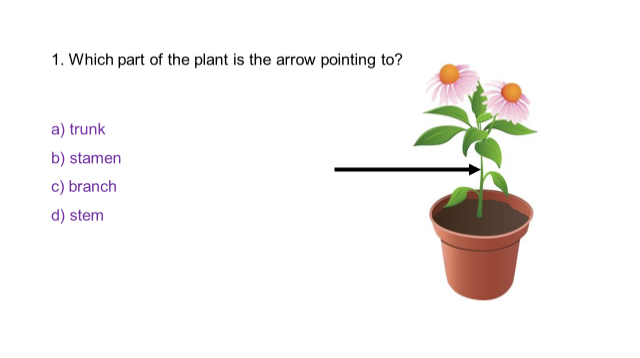

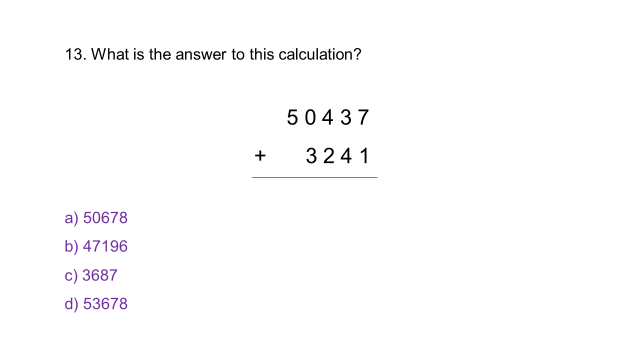

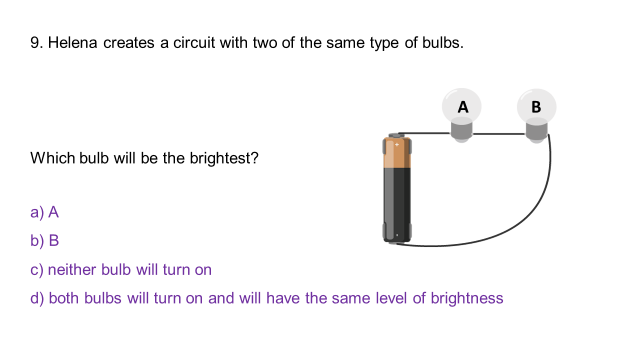

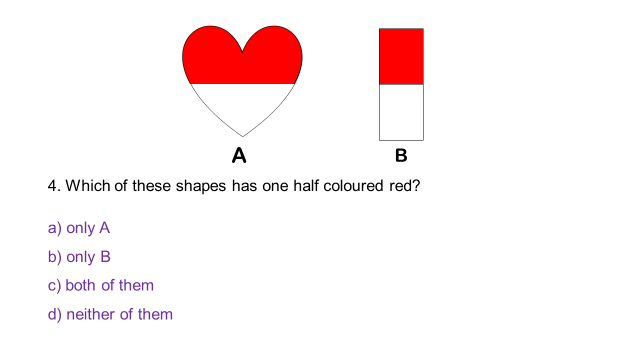


i.

iii.

iv.

ii.

**Figure S1.** Example items from the mathematics and science counterintuitive reasoning task

i. Year 3 mathematics item: Response b) is correct; c) is the intuitive response (i.e. that ‘half’ always refers to a shape split into two parts regardless of the relative size of the two parts); response a) and d) are ‘other’ incorrect (i.e. not the expected intuitive response).ii. Year 5 science item: Response d) is correct; a) is the intuitive response (i.e. that the bulb closest to the energy source will receive more of the electricity/energy and therefore will be brighter than a bulb positioned further away); b) and c) are ‘other’ incorrect. iii. Year 3 science item not related to counterintuitive concept: Response d) is correct, and there is not a common intuitive incorrect response option. iv. Year 5 mathematics item not related to a counterintuitive concept: Response a) is correct, and there is not a common intuitive incorrect response option.

i.

ii.

**Figure S2**. Example items from the Stroop-like chimeric animals task

i. Congruent item: ‘Duck’ is the correct response ii. Incongruent item: ‘Sheep’ is the correct response


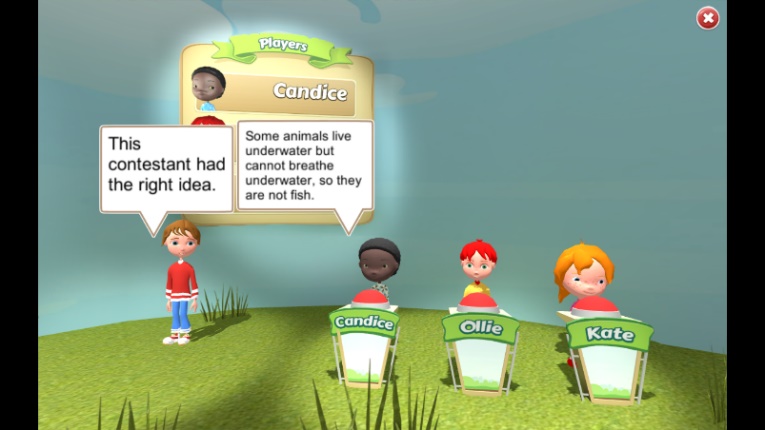

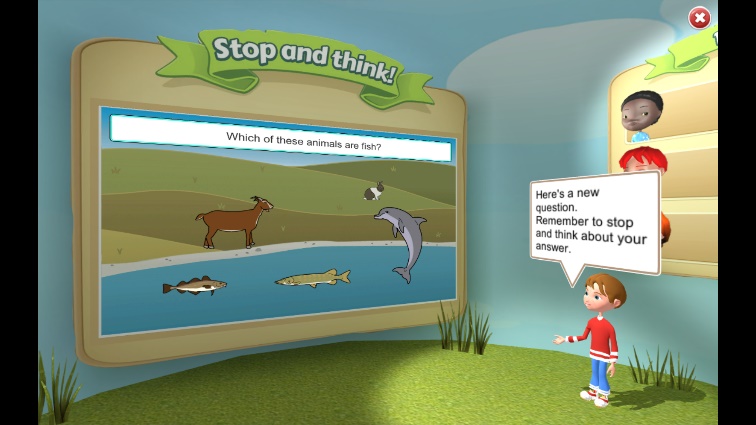

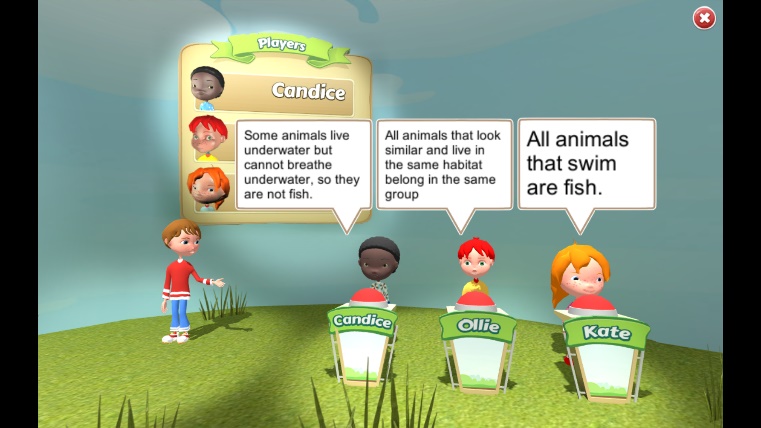

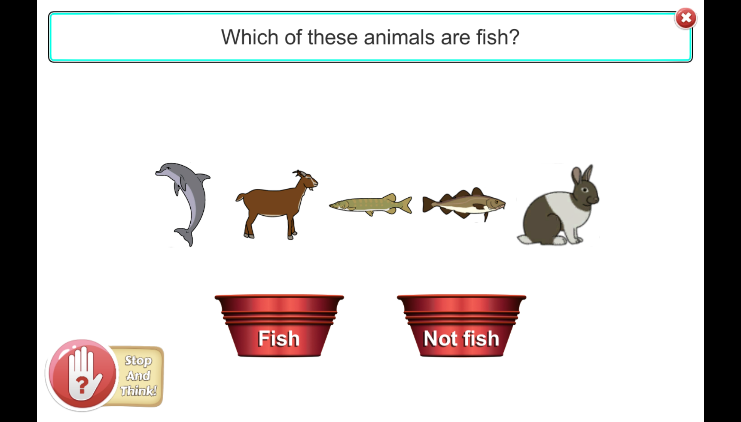


ii.

i.

iv..00

iii..00

**Figure S3.** ‘Stop & Think’ interface and inhibitory control prompts

i. Game show host Andy reminding the user to ‘stop and think’ before responding to the task.ii. Pulsing ‘Stop and Think’ logo (bottom left of screen).iii. Contestants’ presenting their thoughts (reasoning) about the task. In this example, the character Candice has the correct reasoning, Ollie has the intuitive incorrect reasoning, and Kate is more generally incorrect. iii. The contestant with the correct reasoning is revealed.

Correct answer revealed

Attempt 1


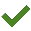


Correct contestant’s reasoning revealed

Attempt 4


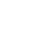

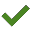


Attempt 3


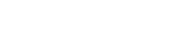

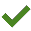


Attempt 2


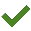

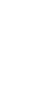

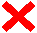

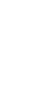

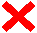

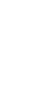

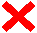

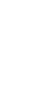

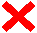


Structured practice

“Have another go”

Contestants’ reasoning

Correct answer revealed

**Figure S4.** Exploratory phase task progression and levels of support

Structured Practice

(up to five subtasks)

Exploratory

(one subtask)

Session

Stop & Think

Subtasks

Mathematics

(6 minutes)

Science

(6 minutes)

30 sessions

(3 sessions x 10 weeks)


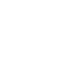

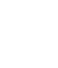


Exploratory

(one subtask)

Structured Practice

(up to five subtasks)

**Figure S5.** Stop & Think sessions structure


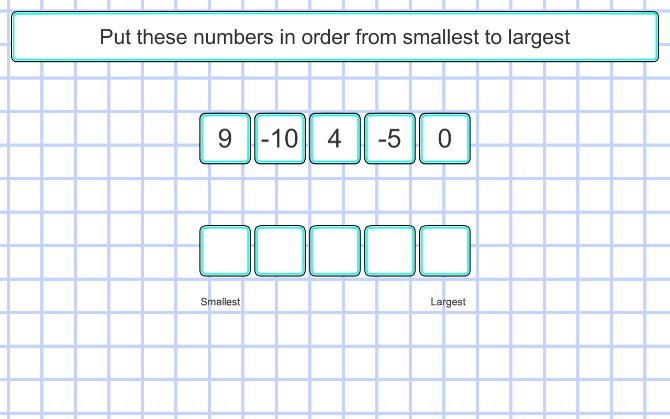

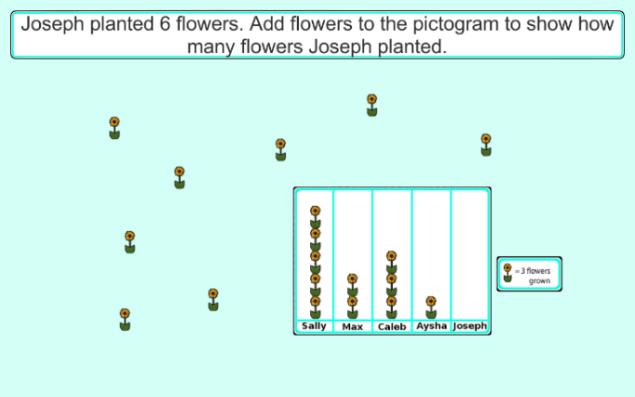

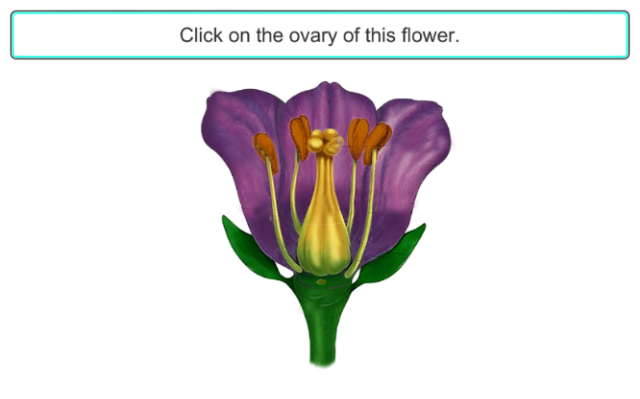

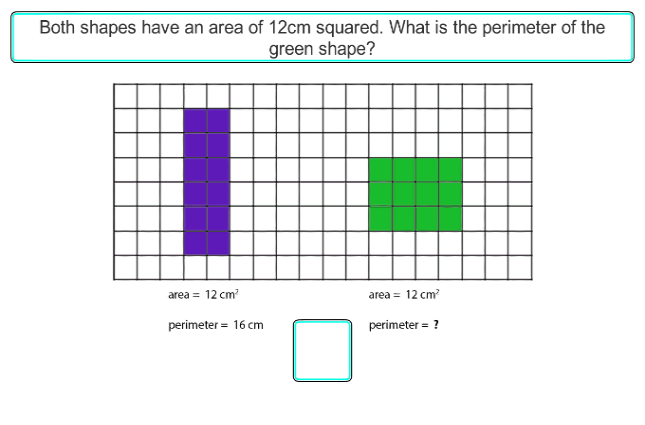

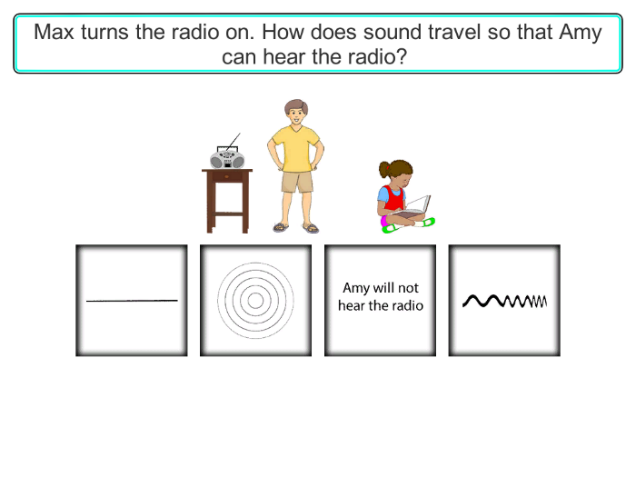

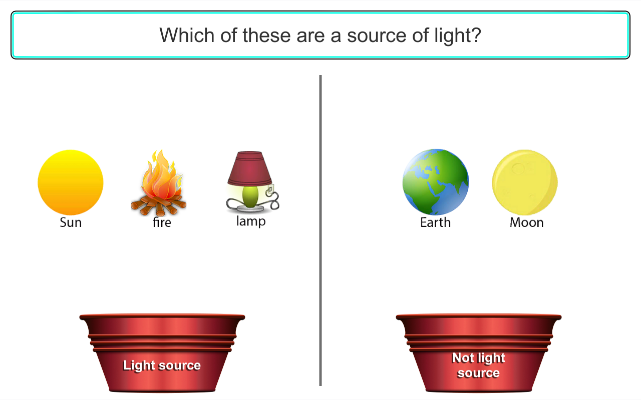

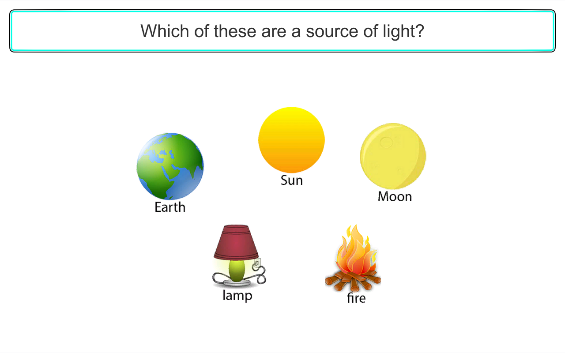


ii.

i.00

iv.

iii.

v.

vi.

**Figure S6.** Stop & Think subtask examples with different response types.

i. Enter, Year 5 Mathematics. Incorrect intuitive reasoning: If the magnitude of the areas of two shapes are identical, the magnitude of the perimeters of the same shapes are also identical. ii. Select, Year 5 Science. Incorrect intuitive reasoning: Sound travels in straight lines. iii. Sort, Year 3 Science. Incorrect intuitive reasoning: The moon is a source of light. iv. Order, Year 5 Mathematics. Incorrect intuitive reasoning: Negative numbers increase in size in the same way positive numbers do (i.e. -5<-10).v. Construct, Year 3 Mathematics. Incorrect intuitive reasoning: In a pictogram, each item always represents one unit.vi. Select, Year 3 Science. No counterintuitive concept.

**Table S1**. Percentage of items with no response^a^ on mathematics and science tasks at T1 and T2 for Year 3 and Year 5 children.

|  | **Counterintuitive Reasoning task** | | **Progress Test in Science** | | **Progress Test in Maths** | |
| --- | --- | --- | --- | --- | --- | --- |
|  | **Year 3** | **Year 5** | **Year 3** | **Year 5** | **Year 3** | **Year 5** |
| **Time 1** | 0.86 | 0.53 | 2.38 | 2.23 | 3.02 | 5.11 |
| **Time 2** | 0.38 | 0.20 | 1.53 | 4.42 | 2.76 | 3.47 |

^a^ Items with no response were scored as 0 to reflect an incorrect response to a mathematics or science question.
